# Positively Charged Nanostructured Lipid Carriers and Their Effect on the Dissolution of Poorly Soluble Drugs

**DOI:** 10.3390/molecules21050672

**Published:** 2016-05-20

**Authors:** Kyeong-Ok Choi, Jaehyeog Choe, Seokjin Suh, Sanghoon Ko

**Affiliations:** Department of Food Science and Technology, Sejong University, 261 Neungdong-ro, Gwangjin-gu, Seoul 143-747, Korea; ko1786@naver.com (K.-O.C.); choohc2002@naver.com (J.C.); sjs007kr@hanmail.net (S.S.)

**Keywords:** poorly water soluble drug, indomethacin, nanostructured lipid nanocarrier, *N*-(2-hydroxy)propyl-3-trimethyl ammonium chitosan chloride, *in vitro* release

## Abstract

The objective of this study is to develop suitable formulations to improve the dissolution rate of poorly water soluble drugs. We selected lipid-based formulation as a drug carrier and modified the surface using positively charged chitosan derivative (HTCC) to increase its water solubility and bioavailability. Chitosan and HTCC-coated lipid particles had higher zeta-potential values than uncoated one over the whole pH ranges and improved encapsulation efficiency. *In vitro* drug release showed that all NLC formulations showed higher *in vitro* release efficiency than drug particle at pH 7.4. Furthermore, NLC formulation prepared with chitosan or HTCC represented good sustained release property. The results indicate that chitosan and HTCC can be excellent formulating excipients of lipid-based delivery carrier for improving poorly water soluble drug delivery.

## 1. Introduction

Recently, poorly water soluble drugs have received increasing interests in the pharmaceutical industry. According to the Biopharmaceutics Classification System (BCS), drugs are categorized into four classes (class I, II, III, and IV) based on their solubility and membrane permeability [1]. For BCS class II drugs, dissolution is a rate-limiting step to their gastrointestinal absorption and bioavailability. The bioavailability of a class II pharmaceutical compound with adequate cell membrane permeability depends on the dissolution of the compound. This has encouraged the pharmaceutical industry to look for new strategies to enhance the dissolution rate of such drugs in order to improve their bioavailability. When a pharmaceutical compound has high permeability but a water solubility of less than 1 μg/mL, conventional drug formulations may be unacceptable. Thus, the development and selection of suitable formulations are crucial to producing successful products for oral administration of BCS class II drugs.

Orally administered drugs that are rapidly absorbed from the gastrointestinal tract and have a short half-life are quickly eliminated from systemic circulation. This is one of the factors that affect the oral bioavailability of some drugs. Various types of sustained release drug formulations have been suggested to allow the slow release of drugs, resulting in increased residence time in the gastrointestinal tract. However, clinical trials often fail to show evidence of acceptable efficacy.

Currently, several strategies have been suggested for improving the solubility of BCS class II drugs, including crystal modification, particle size reduction, pH modification, amorphization, and lipid-based formulations [2]. Lipid-based formulations have been extensively studied, and have shown various advantages such as the ability to release the drug in a controlled and targeted manner; high and enhanced drug content and stability; favorable biodegradability and biocompatibility; versatility of excipients and formulations; and low risk profile [3]. In particular, nanostructured lipid nanocarriers (NLC) are considered an alternative lipid-based carrier system to liposomal vesicles, emulsions, and solid lipid nanoparticles (SLN). However, they also have some drawbacks that need to be overcome for successful delivery to the target site with controlled release of incorporated drugs. Short gastric retention time (GRT), unpredictable short gastric emptying time (GET), and lipolysis in the upper intestine are responsible for reduced bioavailability of drugs upon oral administration [4]. Surface modification of a drug delivery carrier via introduction of a positive charge is hypothesized to overcome the drawbacks of conventional formulations, improving system stability as well as allowing effective, site-specific drug delivery. In general, positively charged delivery carriers regulate the release rate of drugs depending on biological conditions and enhance drug uptake via electrostatic interactions with sialic acid of mucin in the GI tract [5,6].

In pharmaceutical industry, chitosan is often used as a surface modification agent for various drug delivery systems to improve their performance [7,8]. Chitosan and its derivatives have been extensively studied with respect to their potential to improve the performance of drug carriers by enhancing drug loading, absorption, via specific targeting, controlled and sustained release, among others. In particular, the coating of a drug delivery carrier with cationically modified chitosan derivative was demonstrated to have improved the delivery performance of the cationic primary amino group of chitosan [9]. Based on previous reports, we hypothesized that the impregnation of cationically modified chitosan derivative could improve the dissolution rate of poorly water soluble drugs by coating the surface of conventional NLC formulations. The aim of this study was to improve the solubility of poorly water soluble drugs and investigate the possibility of the application of cationically modified chitosan derivative as a surface modification agent to improve the drug delivery performance of lipid nanocarrier. A model drug, indomethacin was incorporated in NLC formulations to improve its water solubility and bioavailability.

## 2. Results and Discussion

### 2.1. Synthesis and Characterization of HTCC

**Figure 1:**
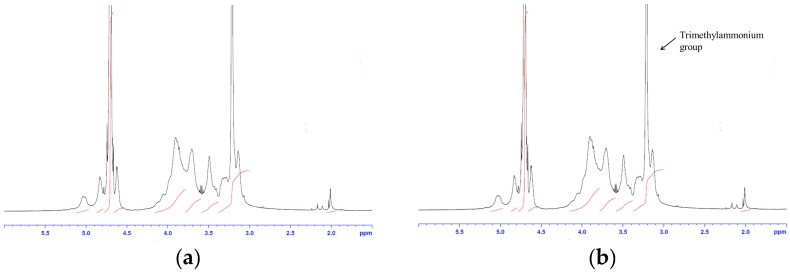
^1^H-NMR spectra of (**a**) chitosan and (**b**) HTCC at 25 °C.

**Figure 2:**
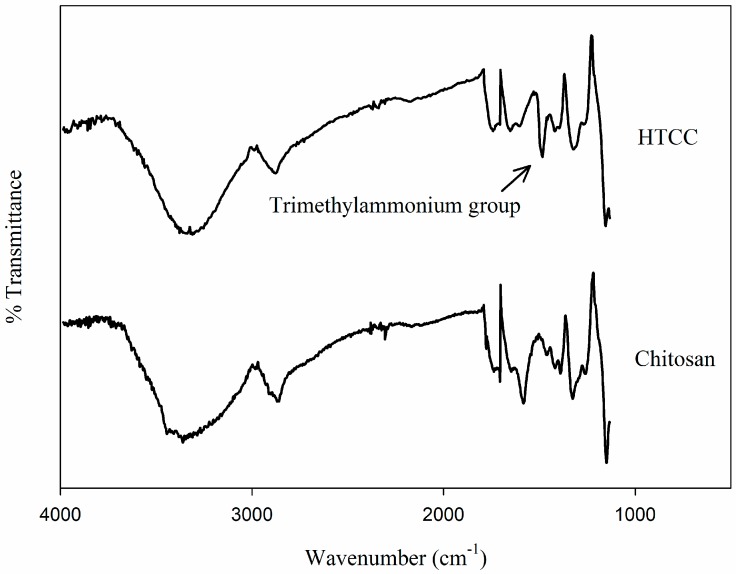
FT-IR spectra of chitosan and HTCC.

HTCC synthesis was confirmed by ^1^H-NMR and FT-IR spectra. The ^1^H-NMR spectra of unmodified chitosan and HTCC are shown in Figure 1. A strong resonance at 3.2 ppm was observed in the HTCC spectrum, indicative of a trimethyl group of the quaternary ammonium side chain; however, this was not observed in the chitosan spectrum [10]. The resonances at around 4.8 ppm and 3.1 ppm were attributable to the H-1 and H-2 of glucosamine unit (GlcN), respectively, in both chitosan and HTCC spectra [11]. Furthermore, the resonance at around 4.6 ppm corresponded to the H-1 of the *N*-acetylglucosamine unit (GlcNAc). The resonance at around 2.0 ppm represents three protons in the acetyl group [12]. The resonances of H-3, 4, 5, and 6 appeared in the range of 3.5–4.0 ppm [12]. The FT-IR spectra shown in Figure 2 also confirmed the successful introduction of GTMAC into the amino group of chitosan. Bands at around 3268, 2882, 1730, 1646, 1580, 1413, 1320, and 1413 cm^−1^ represent N-H, C-H, carbonyl group, primary amide, secondary amide, C-H of secondary amide, and C-N stretching, respectively, which are general characteristic vibrations of GlcN and GlcNAc. A new band at 1475 cm^−1^ in the HTCC spectrum indicates the introduction of trimethylammonium group on the chitosan backbone [13].

**Figure 3:**
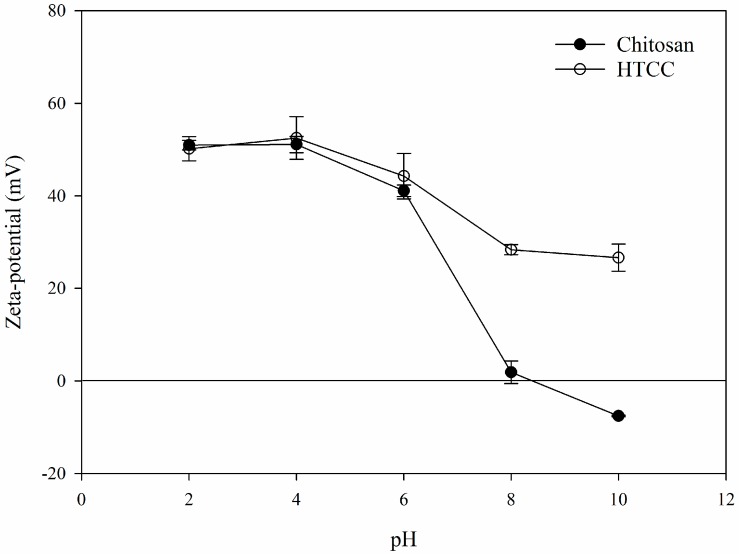
Zeta-potential profiles of chitosan and HTCC as a function of pH.

Zeta-potential profiles of chitosan and HTCC as a function of pH are shown in Figure 3. HTCC showed a highly positive zeta-potential value of +50.98 mV at pH 2. Chitosan also revealed a similar value to HTCC because the amino groups in the chitosan molecules are fully protonated at low pH. However, the zeta-potential value of chitosan decreased below zero at increasing pH. The zeta-potential value of HTCC also decreased with increasing pH; however, this change was more gradual and retained a positive value over the whole pH range. From these results, we concluded that chitosan could be successfully modified with GTMAC to provide permanent positive value, regardless of changes in pH.

### 2.2. Appearance and Encapsulation Efficiency of Nanostructured Lipid Carriers

Different formulations of NLC dispersion were prepared using a hot emulsification method based on phase inversion technique, with the aid of ultrasonic treatment. Compositions of lipid and aqueous phases are represented in Table 1. Blank LNC, indomethacin-loaded NLC, chitosan-coated NLC, and HTCC-coated NLC formulations were abbreviated to NLC_B, NLC_I, NLC/CS_I, and NLC/HTCC_I, respectively. NLC_B formulation was translucent and whitish in appearance. NLC_I formulation was translucent and yellowish, while the NLC/CS_I and NLC/HTCC_I formulations were yellowish, turbid, and milky after preparation. All indomethacin-loaded formulations had a yellowish tinge because indomethacin particle (white) turns bright yellow upon dissolution in lipid phase. After storage for a month, visual observation indicated particle aggregation and precipitation with growing turbidity in NLC_B and NLC_I formulations. In agreement with previous reports that suggested that macromolecular biopolymers stabilize lipid colloids upon storage, chitosan and HTCC seemed to stabilize the surface of the lipid particle to become resistant to inter-particle aggregation [14].

The encapsulation efficiency of three different formulations of indomethacin-loaded NLC was evaluated and the results are represented in Table 1. Encapsulation efficiency of NLC_I formulation was low, despite the high solubility of indomethacin in lipid phase. On the other hand, more drugs were incorporated in NLC/CS_I and NLC/HTCC_I formulations than in the NLC_I formulation. It is assumed that some of the drug molecules would exist at the lipid surface or surfactant layer, so that, the surface-bound drug fraction could easily diffuse out upon dilution and storage. However, for the NLC/CS_I and NLC/HTCC_I formulations, surface coverage with the coating material could prevent the diffusion of surface-bound drug molecules. 

### 2.3. Particle Size Distribution and Zeta-Potential Profiles

Mean particle sizes and polydispersity indexes (PDI) of NLC/CS and NLC/HTCC formulations are listed in Table 2. The mean particle size increased significantly with the addition of chitosan and HTCC, while the PDI value decreased. An increase in mean particle size of NLC/CS and NLC/HTCC formulations seems to relate to their viscosity of the continuous phase. Prior to the preparation of NLC formulation, viscosity values of chitosan and HTCC solutions were 4.8 and 4.2 mPa·s at 80 °C, respectively, which were approximate 4 times higher than that of deionized water. The viscosity of NLC/CS and NLC/HTCC formulations increased after phase inversion. Subsequent ultrasonic treatment increased the viscosities of NLC/CS_I and NLC/HTCC_I formulations up to 87 and 82 mPa·s, respectively. In cases of NLC_B and NLC_I formulations, viscosity increased up to 3.9 and 4.8 mPa·s, respectively, after ultrasonic treatment.

An increase in viscosity of the continuous phase inhibits the mobility and flexibility of surfactant molecules, which retards the adsorption of the surfactant on the surface of newly formed lipid particles. An increase in viscosity of aqueous medium also makes it harder to achieve sufficient acoustic cavitation, which is a powerful driving force for particle reduction during ultrasonic treatment.

**Figure 4:**
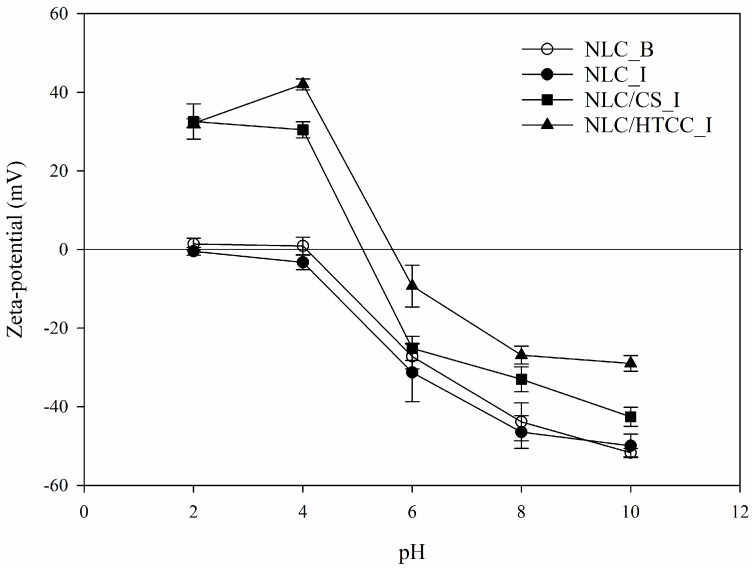
Zeta-potential profiles of different NLC formulations as a function of pH. NLC_B, blank NLC formulation; NLC_I, indomethacin loaded NLC formulation; NLC/CS_I: indomethacin loaded NLC formulation coated with chitosan; NLC/HTCC_I, indomethacin loaded NLC formulation coated with HTCC.

The zeta-potential profiles of the NLC formulations as a function of pH are shown in Figure 4. The formulations of NLC_B and NLC_I showed almost zero mV at pH 2 and 4, which gradually decreased to negative value with increasing pH. The carboxyl groups of oleic acid chains are responsible for the zeta-potentials of NLC_B and NLC_I formulations because carboxyl groups are protonated at low pH and deprotonated at high pH to give a negative zeta-potential value. On the other hand, the formulations of NLC/CS_I and NLC/HTCC_I showed a highly positive zeta-potential value at low pH owing to protonation of the amino group of chitosan and the quaternized ammonium group of HTCC. The zeta-potential values of NLC/CS_I and NLC/HTCC_I also decreased with increasing pH owing to deprotonation of the carboxyl group of oleic acid chain, but more gradually than those of NLC_B and NLC_I formulations.

### 2.4. Differential Scanning Calorimetry

Melting behavior and relative crystallinity of NLC formulations were compared with the bulk lipid material as shown in Table 3. Melting peaks of glyceryl monostearate component for bulk lipid, NLC/HTCC_I, and the other formulations were observed at 58.4, 52.5, and around 47 °C, respectively. As crystallinity of NLC formulations increased, melting point increased as well. It is known that higher crystallinity (highly ordered lipid structure) affects negatively the performance of lipid based carriers such as less encapsulation efficiency and initial burst release *in vitro* [15]. However, in this study, interestingly NLC/HTCC_I formulation showed relatively high crystallinity compared to the other formulations, which seems not to affect the encapsulation efficiency and *in vitro* drug release. Although NLC/HTCC_I formulation was higher in crystallinity, it still provides enough space for entrapment of guest molecules. In addition, higher encapsulation efficiency and less initial drug burst were observed in NLC/HTCC_I formulation compared to the other formulations during *in vitro* test.

**Figure 5:**
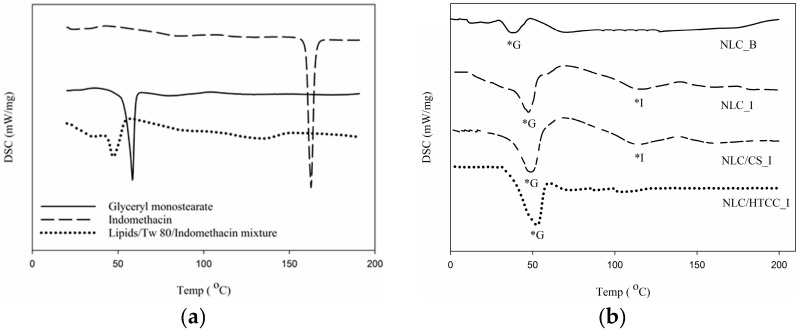
Endothermic peaks of (**a**) bulk glyceryl monostearate, indomethacin, and their mixture and (**b**) NLC formulations; *G, endothermic peak of glyceryl monostearate; *I, endothermic peak of indomethacin.

Endothermic curves of bulk lipid, indomethacin, and their mixture are shown in Figure 5a. Melting peaks of bulk lipid and indomethacin appeared at 58.4 and 162.7 °C, respectively. The mixture of lipid phase and indomethacin showed relatively low melting point and low enthalpy compared to individual lipid or indomethacin since melting and subsequent cooling induced the recrystallization between lipid and indomethacin resulting in the change in the crystalline state [16]. Melting peaks of NLC formulations are shown in Figure 5b. The melting points and enthalpy values were relatively low compared to bulk lipid owing to the same reason in the mixture. Melting peak did not appear in NLC/HTCC_I formulation, but tiny peaks were observed in NLC_I (116.9 °C) and NLC/CS_I (114.4 °C) formulations.

### 2.5. In vitro Drug Release

**Figure 6:**
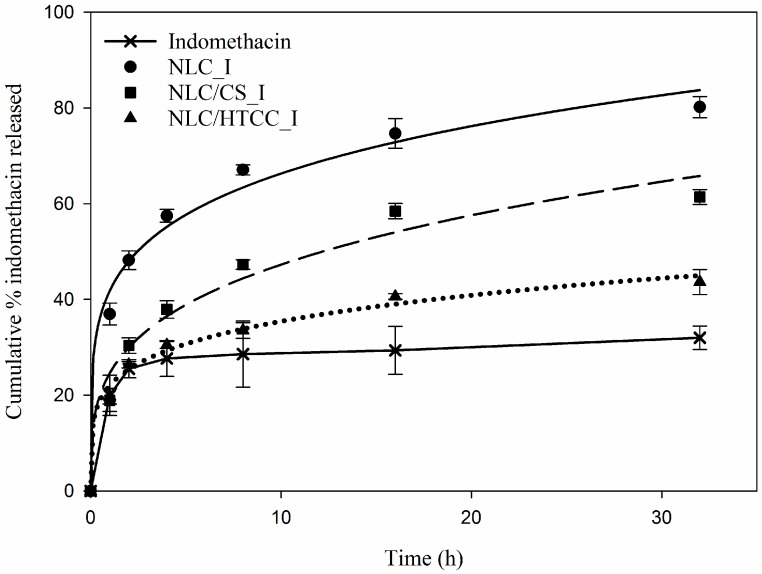
*In vitro* release profiles of three different indomethacin loaded NLC formulations. NLC_I, indomethacin loaded NLC formulation; NLC/CS_I: indomethacin loaded NLC formulation coated with chitosan; NLC/HTCC_I, indomethacin loaded NLC formulation coated with HTCC. Each formulation was fitted according to Korsmeyer-Peppas regression model except pure indomethacin.

Figure 6 shows *in vitro* release profiles of three different indomethacin loaded NLC formulations. The release rate of indomethacin from three different NLC formulations varied depending on the formulations for the carriers. For the all formulations tested in the PBS buffer, indomethacin released fast at the initial period of the *in vitro* release test but subsequently released slowly over time. The order of drug release rate in the formulations was found to decrease in the following order NLC_I > NLC/CS_I > NLC/HTCC_I > drug for 32 h. NLC formulations coated with chitosan and HTCC showed intermediate drug release rate; the cationic surface modification of lipid carrier using chitosan and HTCC provided sustained release characteristics for the indomethacin loaded lipid carriers. It is expected that chitosan and HTCC coated lipid carrier could enhance dissolution rate of drug thanks to the sustained release behavior. In addition, the mucoadhesive property of chitosan and HTCC contributes to the increase in bioavailability of drug since residence time was prolonged in the gastrointestinal track.

*In vitro* release profiles of indomethacin loaded NLC formulations were fitted using various non-linear regression models such as zero order, first order, Higuchi, and Korsmeyer-Peppas equations as shown in Figure 6. In addition, the regression coefficient (R^2^) and release exponent (n) values calculated using the non-linear regression models for the formulations are listed in Table 4. All NLC formulations were fitted well with Korsmeyer-Peppas model since R^2^ values of NLC_I, NLC/CS_I and NLC/HTCC_I were 0.955, 0.9372, and 0.9645, respectively. The n value (<0.43) of Korsmeyer-Peppas equation indicated that the release behavior of all formulations followed Fickian diffusion mechanism.

### 2.6. Static In vitro Digestion

**Figure 7:**
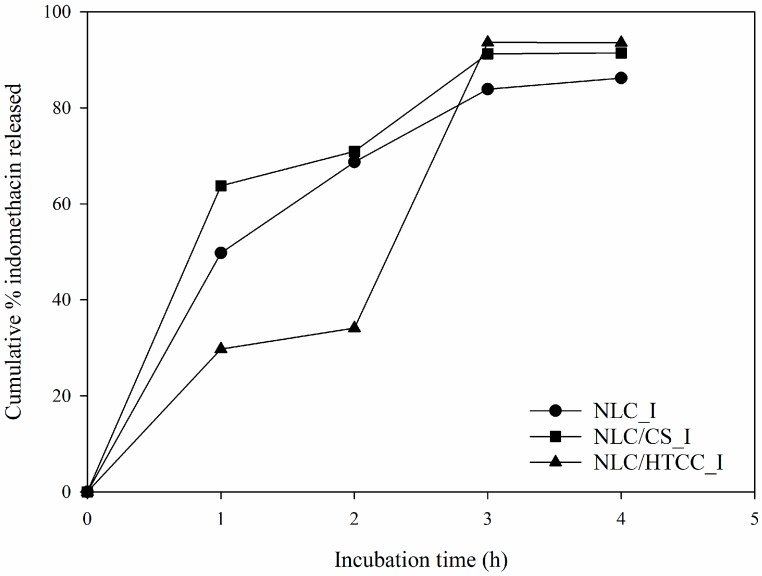
*In vitro* digestion characteristics of three different indomethacin loaded NLC formulations. NLC_I, indomethacin loaded NLC formulation; NLC/CS_I: indomethacin loaded NLC formulation coated with chitosan; NLC/HTCC_I, indomethacin loaded NLC formulation coated with HTCC.

*In vitro* drug release characteristics of indomethacin with the presence of digestive enzymes were measured in static, simulated gastric and intestinal fluids for 4 h (2 h each), and the results are shown in Figure 7. For NLC_I and NLC/CS_I formulations, release rates of indomethacin from lipid particles were relatively fast in the simulated gastric fluid and slow in the intestinal fluid. We also observed that more than 60% of entrapped indomethacin was released from lipid nanoparticles in the gastric fluid. NLC/HTCC_I formulation showed a relatively slow release rate of indomethacin (approximately 30% within 2 h), and similar release characteristics to the other formulations in the simulated intestinal fluid. From the theoretical point of view, rapid release of drug entrapped in lipid nanoparticle should take place in the intestinal fluid in which lipolysis occurs. In this study, however, significant amounts of indomethacin were released in the gastric fluid. Thus, we assumed that a significant amount of indomethacin was distributed on the surface of the lipid particle or the interface between hydrophobic region of lipid and the surfactant layer during crystallization of the lipid particle. This surface-bound indomethacin could simply diffuse out into aqueous medium during digestion. For NLC/HTCC_I formulation, HTCC with positively charged alkyl chains on the surface of the lipid particle seemed to prevent the diffusion of indomethacin molecules, thus retarding the release of the drug in the gastric fluid.

## 3. Experimental Section

### 3.1. Synthesis and Characterization of HTCC

Chitosan (Mw 600,000–800,000, DD 90%) and glycidyl trimethylammonium chloride (GTMAC) were purchased from Sigma-Aldrich (St. Louis, MO, USA) and used to synthesize HTCC. *N*-(2-Hydroxy)propyl-3-trimethyl ammonium chitosan chloride (HTCC) was prepared by reacting chitosan with GTMAC in acetic aqueous solution (2% *v*/*v*) [17]. Chitosan powder (6 g) was dissolved in 500 mL of acetic aqueous solution under magnetic stirring at 60 °C. Following complete dissolution of chitosan particles, the temperature was raised to 85 °C with constant stirring. GTMAC was added to the chitosan solution dropwise in five portions at 3-h intervals (7.3 mL each). The total reaction time was 18 h from the first addition of GTMAC. The reaction mixture was then cooled to room temperature and diluted with ethanol. HTCC was precipitated by pouring the reaction mixture in acetone. The precipitate was isolated by centrifugation and washed by ethanol, acetone and finally, diethyl ether. The final product was dried and kept in a desiccator until use. 

FT-IR spectra were recorded on a Nicolet 380 FT-IR Spectrometer (Thermo Fisher Scientific Inc., Waltham, MA, USA). ^1^H-NMR spectra were obtained using a Bruker Avance II 500 spectrophotometer (Karlsruhe, Germany) at room temperature. The data was collected with 128 scans. NMR samples were prepared by dissolving chitosan and HTCC powder in D_2_O containing a drop of DCl. Zeta potentials were measured using a zeta-potential analyzer (Delsa nano C, Beckman Coulter, Inc., Fullerton, CA, USA) at a pH range of 2–10. 

### 3.2. Solubility Test of Indomethacin in Lipid Phase

Liquid oleic acid and solid glyceryl monostearate were used as components of lipid matrix and Tween 80 was used as a surfactant. They were purchased from Sigma-Aldrich. Indomethacin was used as a model drug and purchased from Sigma-Aldrich. Chitosan and HTCC prepared as described above, were used for surface modification of lipid nanoparticles.

The solubility of indomethacin in lipid phase consisting of oleic acid, glyceryl monostearate, and Tween 80 (1:1:1, *w*/*w*/*w*) was determined prior to encapsulation. Indomethacin (100 mg) was dissolved in 1 g of lipid phase at 80 °C for 20 min and cooled to room temperature. The lipid phase was centrifuged at 8000 rpm for 2 min at 40 °C to remove undissolved drug particles. The supernatant was separated carefully from the sediment and dissolved in DMSO at 60 °C. After cooling, the solution was centrifuged at 8000 rpm for 30 min at 4 °C to separate the oil phase. The DMSO layer was carefully transferred to a cuvette and the absorbance was measured at a wavelength of 320 nm on a spectrophotometer (DU 730, Beckman Coulter, Inc., Fullerton, CA, USA). A calibration curve was prepared from the indomethacin standard solutions (3, 5, 10, 20, 30, 40, 50 μg/mL in DMSO). The concentration of indomethacin in the lipid phase was calculated from calibration curve (R^2^ = 0.9993).

### 3.3. Preparation of Nanostructured Lipid Carriers

Four different formulations of nanostructured lipid carrier were prepared by a two-step emulsification method. In the first step, coarse emulsion was prepared by phase inversion emulsification. Lipid phase (3%) and 48.5 mL of aqueous phase were heated to 80 °C separately, and the aqueous phase was added dropwise to the lipid phase at a constant rate (5 mL/min) using a peristaltic pump (Longer Precision Pump Co. Ltd., Hebei, China) under magnetic stirring. For NLC/CS_I and NLC/HTCC_I formulations, 0.1% chitosan and HTCC were dissolved directly in the aqueous phase. The coarse emulsion was further stirred for 10 min. In the second step, the coarse emulsion was treated with a probe ultrasonic processor (VCX 750, Sonics & Materials, Inc., Newtown, CT, USA) at 35% amplitude for 20 min. The hot nanoemulsion was cooled to room temperature in order to induce crystallization. 

### 3.4. Particle Size Measurement

Hydrodynamic particle diameters were measured using a particle size analyzer (Delsa nano C, Beckman Coulter, Inc.). Mean particle size and polydispersity index were recorded based on intensity-based distribution. Each sample was measured in triplicate with three different sets at 25 °C and a scattering angle of 165°.

### 3.5. Zeta-Potential Measurement

Zeta-potential was measured to determine surface charge distributions as a function of pH. Prior to measurement, 10 mL of LNC dispersions was washed with ethanol and water three times by repeated centrifugation and resuspension. The final precipitates were redispersed in 10 mL of deionized water by ultrasonic treatment at 30% amplitude for 10 min. Each sample was diluted in deionized water and subjected to a pH gradient from 2 to 10. Then, the diluted sample was injected into a flow cell and the zeta-potential was measured at different cell positions, as follows: 0.7, 0.35, 0, −0.35 and −0.7. Each sample was measured in triplicate with three different sets at 25 °C and a scattering angle of 35° using a zeta-potential analyzer.

### 3.6. Encapsulation Efficiency

Encapsulation efficiency and drug loading were determined by measuring the concentration of indomethacin loaded in the lipid carrier. A 1 mL aliquot of NLC dispersion was added to 4 mL of DMSO and stirred at 60 °C in order to completely dissolve the lipid particle and collect the released indomethacin. The mixture was then cooled and filtered through a 0.2 μm syringe filter. An HPLC system (Agilent Technologies, Santa Clara, CA, USA) equipped with an auto sampler and U*V*/*V*is detector was used to determine the concentration of indomethacin. The chromatograms of indomethacin were recorded using a Unisol reversed-phase C18 column (4.6 mm × 250 mm, 5 μm, 100 Å) (Agela Technologies, Wilmington, DE, USA) with a U*V*/*V*is detector at 320 nm. The flow rate was 1 mL/min and the mobile phase consisted of acetonitrile and 0.02 M acetate buffer (60:40, *v*/*v*). A calibration curve (R^2^ = 0.9998) was prepared from indomethacin standard solutions (10, 50, 100, 300, 500, 700 and 1000 μg/mL). Encapsulation efficiency was calculated using the following equation [18].

(1) $$\text{Encapsulation efficiency }\left(\text{\%}\right)=\;\frac{\text{Total drug content enprapped}}{\text{Initial drug content}}\;\times 100$$

### 3.7. Differential Scanning Calorimetry

Melting behaviors and relative crystallinity of the NLC formulations were determined by differential scanning calorimetry (DSC 200 F3, Netzsch-Gerätebau GmbH, Selb, Germany). For the DSC measurement, exactly 3 mg of the lyophilized NLC formulations were weighed in a standard aluminum pan, and hermetically sealed with a standard aluminum lid. An empty pan was used as a reference. All samples were heated and cooled from 0 to 200 °C at a scan rate of 5 °C/min. Melting temperature and relative crystallinity of the formulations were determined from the melting curve based on the temperature at which endothermic peak appeared and the integration of the peak, respectively. Crystallinity was calculated by the manufacturer’s instructions (Netzsch-Gerätebau GmbH). In this study, crystallization enthalpy of all NLC formulations was negligible.

(2) $$\text{Relative crystallinity }\left(\text{\%}\right)=\;\frac{\text{Integration of melting enthalpy of NLC}}{\text{Integration of melting enthalpy of bulk lipid}}\times 100$$

### 3.8. In vitro Drug Release Test

*In vitro* release test of indomethacin loaded NLC carriers was carried out for NLC_I, NLC/CS_I and NLC/HTCC_I formulations. Briefly, 5 mL of aqueous NLC formulations equivalent to 4.2, 5.3 and 5.6 mg indomethacin for NLC_I, NLC/CS_I and NLC/HTCC_I, respectively, were introduced in 10 mM PBS (pH 7.4, Sigma-Aldrich) solution in 250 mL flask. Additionally, drug release test on non-encapsulated indomethacin (7.4 mg) was performed as a control group. The flasks with 100 mL of *in vitro* release test solutions were incubated at 37 °C on a temperature controlled hot plate with constant stirring at 300 rpm for 36 h. Five mL aliquot was collected from each flask at predetermined time intervals using a syringe with a 0.45 μm pre-filter. After collecting each aliquot, 5 mL of fresh PBS buffer solution was added to the flask. The collected sample solution was filtered using 0.22 μm syringe filter again and transferred to a cuvette. Absorbance was measured at a wavelength of 298 nm using a spectrophotometer. A calibration curve (R^2^ = 0.9966) was plotted using the absorbance data measured at indomethacin concentrations (1, 3, 5, 10, 30, 50, and 100 μg/mL in 10mM NaOH). *In vitro* drug release tests were performed in triplicate for each sample. *In vitro* release profiles were fitted using several non-linear models such as zero-order (cumulative percentage of drug released *vs.* time), first-order (log cumulative percentage of drug remaining *vs.* time), Higuchi (cumulative percentage of released *vs.* square root of time), and Korsmeyer-Peppas (log cumulative percentage of drug released *vs.* log time) equations.

### 3.9. Static in Vitro Digestion Test

The indomethacin release properties of lipid nanoparticles were determined by a static *in vitro* digestion model described by Minekus *et al.* [17]. Digestion was initiated by mixing 10 mL of NLC dispersion with 9 mL of simulated gastric fluid (SGF) containing electrolytes and pepsin (EC 3.4.23.1) (6.9 mM KCl, 9.0 mM KH_2_PO_4_, 12.5 mM NaHCO_3_, 11.8 mM NaCl, 0.4 mM MgCl_2_(H_2_O)_6_, 0.5 mM (NH_4_)_2_CO_3_, 0.15 mM CaCl_2_(H_2_O)_2_, 2000 U/mL pepsin). The pH of the mixture was adjusted to 3 by 1N HCl solution, and the resulting mixture was made up to 20 mL using deionized water. The mixture was incubated at 37 °C under constant magnetic stirring at 300 rpm for 2 h. After digestion in SGF, 10 mL of gastric chyme was mixed with 9 mL of simulated intestinal fluid (SIF) containing appropriate electrolyte concentrations, pancreatin (EC 232-468-9,4 USP specifications), and bile extract (bile extract porcine, Sigma-Aldrich) (6.8 mM KCl, 0.8 mM KH_2_PO_4_, 85 mM NaHCO_3_, 38.4 mM NaCl, 0.33 mM MgCl_2_(H_2_O)_6_, 0.6 mM CaCl_2_(H_2_O)_2_, 800 U/mL pancreatin, 300 mg bile extract porcine), and incubated for a further 2 h. During the digestion, 1 mL of reaction mixture was withdrawn and the same volume of fresh SGF or SIF solution was added every 1 h. Free indomethacin molecules were collected by ultrafiltration (Vivaspin20 1000K device, MWCO 1,000 K, Sartorius Stedim Lab Ltd., Gloucestershire, UK). The amount of free indomethacin concentration was measured by HPLC described in the previous section.

## 4. Conclusions

The present research paper proposed cationically modified lipid-based drug carriers (NLCs) for improving water solubility and bioavailability of poorly soluble drug. Indomethacin was selected as a model drug and incorporated in NLCs. Positively charged chitosan derivative, HTCC was synthesized to modify the surface of NLCs cationically. NLC/CS_I and NLC/HTTC_I formulations showed higher encapsulation efficiency for indomethacin than NLC_I formulation. NLC/CS_I and NLC/HTCC_I formulations are considered as effective carriers for sustained drug release. Thus, it is concluded that positively charged NLCs such as NLC/CS_I and NLC/HTCC_I formulations could improve bioavailability of poorly soluble drugs.

## Figures and Tables

**Table 1:** Compositions to prepare indomethacin loaded NLC formulations.

Formulation	Solid Lipid (%)	Liquid Lipid (%)	Tween 80 (%)	Coating Material (%)	Viscosity of Aqueous Phase (mPa·s)	Indomethacin (mg)
NLC_B	1	1	1	-	0.36	-
NLC_I	1	1	1	-	0.36	74
NLC/CS_I	1	1	1	0.1	4.8	74
NLC/HTCC_I	1	1	1	0.1	4.2	74

NLC_B, blank NLC formulation; NLC_I, indomethacin loaded NLC formulation; NLC/CS_I, indomethacin loaded NLC formulation coated with chitosan; NLC/HTCC_I, indomethacin loaded NLC formulation coated with HTCC. Viscosity values were measured at 80 °C.

**Table 2:** Particle size distribution and encapsulation efficiency of indomethacin loaded NLC formulations.

Formulation	Particle Size Distribution		Encapsulation Efficiency (%)	Drug Loading (%)
	Mean Particle Size (nm)	Polydispersity Index		
NLC_B	255.6 ± 2.4 ^b^	0.268 ± 0.002 ^a^	-	-
NLC_I	277.0 ± 22.9 ^b^	0.270 ± 0.015 ^a^	56.8 ± 0.5 ^c^	4.2 ± 0.04 ^c^
NLC/CS_I	360.2 ± 34.1 ^a^	0.247 ± 0.026 ^b^	72.2 ± 1.9 ^b^	5.3 ± 0.13 ^b^
NLC/HTCC_I	351.9 ± 22.4 ^a^	0.238 ± 0.02 ^b^	74.4 ± 0.7 ^a^	5.5 ± 0.05 ^a^

NLC_B, blank NLC formulation; NLC_I, indomethacin loaded NLC formulation; NLC/CS_I, indomethacin loaded NLC formulation coated with chitosan; NLC/HTCC_I, indomethacin loaded NLC formulation coated with HTCC. Letters in the same column indicates significant difference at *p* < 0.05.

**Table 3:** Melting parameters and relative crystallinity of indomethacin loaded NLC formulations.

Formulation	Melting Temp. (°C)	Enthalpy (J/mg)	Relative Crystallinity (%)
NLC_B	47.4 ± 0.9 ^b^	31.9 ± 1.6 ^b^	30.0 ± 069 ^b^
NLC_I	46.8 ± 0.5 ^b^	31.8 ± 4.3 ^b^	30.0 ± 0.0 ^b^
NLC/CS_I	47.8 ± 2.1 ^b^	31.0 ± 2.3 ^b^	29.2 ± 2.1 ^b^
NLC/HTCC_I	52.4 ± 0.5 ^a^	39.0 ± 0.5 ^a^	36.7 ± 0.4 ^a^

NLC_B, blank NLC formulation; NLC_I, indomethacin loaded NLC formulation; NLC/CS_I, indomethacin loaded NLC formulation coated with chitosan; NLC/HTCC_I, indomethacin loaded NLC formulation coated with HTCC. Different letters in the same column indicate a significant difference (*p* < 0.05).

**Table 4:** Regression coefficient (R^2^) and release exponent (n) values calculated using the non-linear regression models for *in vitro* release profiles of indomethacin loaded NLC formulations.

Formulation	Zero Order R^2^	First Order R^2^	Higuchi R^2^	Korsmeyer-Peppas R^2^ (*n*)
NLC_I	0.7319	0.8545	0.8822	0.9555 (0.2202)
NLC/CS_I	0.7405	0.8126	0.8894	0.9372 (0.3312)
NLC/HTCC_I	0.7894	0.8263	0.9172	0.9645 (0.2200)

NLC_B, blank NLC formulation; NLC_I, indomethacin loaded NLC formulation; NLC/CS_I, indomethacin loaded NLC formulation coated with chitosan; NLC/HTCC_I, indomethacin loaded NLC formulation coated with HTCC. *n* value indicates release exponent.

## References

[1] Arrunategui L.B., Silva-Barcellos N.M., Bellavinha K.R., Ev L.S., de Souza J. (2015). Biopharmaceutics classification system: importance and inclusion in biowaiver guidance. Braz. J. Pharm. Sci..

[2] Kawabata Y., Wada K., Nakatani M., Yamada S., Onoue S. (2011). Formulation design for poorly water-soluble drugs based on biopharmaceutics classification system: Basic approaches and practical applications. Int. J. Pharm..

[3] Shrestha H., Bala R., Arora S. (2014). Lipid-based drug delivery systems. Int. J. Pharm..

[4] Nayak A.K., Maji R., Das B. (2010). Gastroretentive drug delivery systems: A review. Asian J. Pharm. Clin. Res..

[5] Kobayashi K., Wei J., Iida R., Ijiro K., Niikura K. (2014). Surface engineering of nanoparticles for therapeutic applications. Polym. J..

[6] Mishra G.P., Bagui M., Tamboli V., Mitra A.K. (2011). Recent applications of liposomes in ophthalmic drug delivery. J. Drug Deliv..

[7] Wang Y., Li P., Kong L. (2013). Chitosan-modified PLGA nanoparticles with versatile surface for improved drug delivery. AAPS PharmSciTech.

[8] Ramalingam P., Ko Y. (2015). Enhanced oral delivery of curcumin from *N*-trimethyl chitosan surface-modified solid lipid nanoparticles: Pharmacokinetic and brain distribution evaluations. Pharm. Res..

[9] Sashiwa H., Aiba S. (2004). Chemically modified chitin and chitosan as biomaterials. Prog. Polym. Sci..

[10] Wu J., Su Z.G., Ma G.H. (2006). A thermo- and pH-sensitive hydrogel composed of quaternized chitosan/glycerophosphate. Int. J. Pharm..

[11] Hirai A., Odani H., Nakajima A. (1991). Determination of degree of deacetylation of chitosan by 1H NMR spectroscopy. Polym. Bull..

[12] Varum K.M., Antohonsen M.W., Grasdalen H., Smidsrød O. (1991). Determination of the degree of *N*-acetylation and the distribution of *N*-acetyl groups in partially *N*-deacetylated chitins (chitosans) by high-field n.m.r. spectroscopy. Carbohydr. Res..

[13] Yang X., Zhang C., Qiao C., Mu X., Li T., Xu J., Shi L., Zhang D. (2015). A simple and convenient method to synthesize *N*-[(2-hydroxyl)-propyl-3-trimethylammonium] chitosan chloride in an ionic liquid. Carbohydr. Polym..

[14] Desbrieres J., Bousquet C., Babak V. (2010). Surfactant-chitosan interactions and application to emulsion stabilization. Cell. Chem. Technol..

[15] Montasser I., Shahgaldian P., Perret F., Coleman A. (2013). Solid lipid nanoparticle-based calix[n]arenes and calix-resorcinarenes as building blocks: Synthesis, formulation and characterization. Int. J. Mol. Sci..

[16] Choi K.O., Aditya N.P., Ko S. (2014). Effect of aqueous pH and electrolyte concentration on structure, stability and flow behavior of non-ionic surfactant based solid lipid nanoparticles. Food Chem..

[17] Lim S.H., Hudson S.M. (2004). Synthesis and antimicrobial activity of a water-soluble chitosan derivative with a fiber-reactive group. Carbohydr. Res..

[18] Papadimitriou S., Bikiaris D. (2009). Novel self-assembled core–shell nanoparticles based on crystalline amorphous moieties of aliphatic copolyesters for efficient controlled drug release. J. Control. Release.

